# Hydrogen Gas Sensor Based on Nanocrystalline SnO_2_ Thin Film Grown on Bare Si Substrates

**DOI:** 10.1007/s40820-015-0057-1

**Published:** 2015-08-19

**Authors:** Imad H. Kadhim, H. Abu Hassan, Q. N. Abdullah

**Affiliations:** 1 Nano-Optoelectronics Research and Technology Laboratory, School of Physics, Universiti Sains Malaysia, 11800 Gelugor, Penang Malaysia; 2 Ministry of Education, Baghdad, Iraq; 3 Physics Department, College of Education, Tikrit University, Tikrit, Iraq

**Keywords:** SnO_2_, Glycerin, Sol–gel, Schottky contact, Hydrogen sensor

## Abstract

In this paper, high-quality nanocrystalline SnO_2_ thin film was grown on bare Si (100) substrates by a sol–gel method. A metal–semiconductor–metal gas sensor was fabricated using nanocrystalline SnO_2_ thin film and palladium (Pd) metal. The contact between Pd and nanocrystalline SnO_2_ film is tunable. Ohmic barrier contact was formed without addition of glycerin, while Schottky contact formed by adding glycerin. Two kinds of sensor devices with Schottky contact were fabricated (Device 1: 8 h, 500 °C; Device 2: 10 h, 400 °C). The room temperature sensitivity for hydrogen (H_2_) was 120 and 95 % in 1000 ppm H_2_, and the low power consumption was 65 and 86 µW for two devices, respectively. At higher temperature of 125 °C, the sensitivity was increased to 195 and 160 %, respectively. The sensing measurements were repeatable at various temperatures (room temperature, 75, 125 °C) for over 50 min. It was found that Device 1 has better sensitivity than Device 2 due to its better crystallinity. These findings indicate that the sensors fabricated on bare Si by adding glycerin to the sol solution have strong ability to detect H_2_ gas under different concentrations and temperatures.

## Introduction

Tin dioxide (SnO_2_) has attracted lots of attentions in wide applications such as the detection of inflammable gases, volatile organic compounds, and toxic gases, due to its unique physical and chemical properties. SnO_2_ is an n-type semiconductor with tetragonal rutile structure and large energy band gap of 3.6 eV at 300 K [1]. Till now, there are many different methods to prepare SnO_2_ thin film, for example, sol–gel [2], thermal evaporation [3], chemical vapor deposition (CVD) [4], radio frequency (RF) magnetron sputtering [5], and spray pyrolysis [6]. Among these methods, the sol–gel method has been widely investigated because of its many advantages such as low reaction temperature, low cost, and easy process [1]. However, Imad et al. [7] found that the sol–gel method without adding glycerin lead to produce thin films suffered from the formation of cracks.

Hydrogen (H_2_) is a kind of more efficient and clean source of energy which has been used in automobiles, aircraft, fuel cells, and chemical industries, etc. [8, 9]. Since H_2_ gas is colorless, odorless, highly volatile, and inflammable [9], the detection at room temperature (RT) is very important for chemical industries and environmental applications. Detection sensor is a usual method to alarm the formation of potentially explosive mixtures of H_2_ in air ambient and therefore to prevent the risk of explosions and fires [10–12]. RT H_2_ gas sensor also attracts much attention in other fields because of their particularly low power consumption [13], the ability to be used safely in flammable environments [14], and long lifetime [15].

Recently, gas sensing mechanism for SnO_2_ films has been studied for different periods [16]. Efforts have also been done for verifying their RT detection by using nano-sized SnO_2_ and applying dopants in the SnO_2_ thin films [17]. Usually, the gas sensing tests were carried out at high operation temperature [18]. Hamaguchi et al. [19] reported that the H_2_ gas sensor can not be performed at low temperature and therefore to extend the response and recovery time.

This paper focuses on fabrication of functional nanocrystalline SnO_2_ thin films and performance of H_2_ gas sensors at RT for different gas concentrations. The main goal of this study is to take the advantage of adding glycerin to the sol solution to solve the cracks problem of nanocrystalline SnO_2_ thin films on bare Si (100) substrates, and then to fabricate thin film gas sensor that could emerge reasonable sensitivity.

## Experimental

The p-type (100) silicon wafer (10 mm × 10 mm) was cleaned by the standard Radio Corporation of America (RCA) method. Nanocrystalline SnO_2_ thin films were grown using sol–gel spin coating method [2, 20]. 0.1 M tin (II) chloride dihydrate (SnCl_2_·2H_2_O) was dissolved via 70 mL of pure ethanol (C_2_H_5_OH) and placed in covered flasks. The resulted sol solutions placed in closed flasks and stirred by magnetic stirrers for 3 h and kept at 70 °C for 8 and 10 h, respectively. Moreover, glycerin (C_3_H_8_O_3_) was added to a volume ratio of 1:12 in order to eliminate cracks [7]. The process of preparing the sol solutions was separately completed at RT for the remainder of the 24 h. Thereafter, the sol solutions were spin-coated on Si (100) substrates at a rotation speed of 3000 rpm for 30 s. The as-deposited films were oven-dried at 100 °C for 10 min, to obtain high thickness, spin coating and drying operations were repeated 10 times for all samples at different aging heat times. The whole samples were annealed at 400 and 500 °C in air ambient for 2 h in order to achieve the crystallization of SnO_2_.

**Fig. 1:**
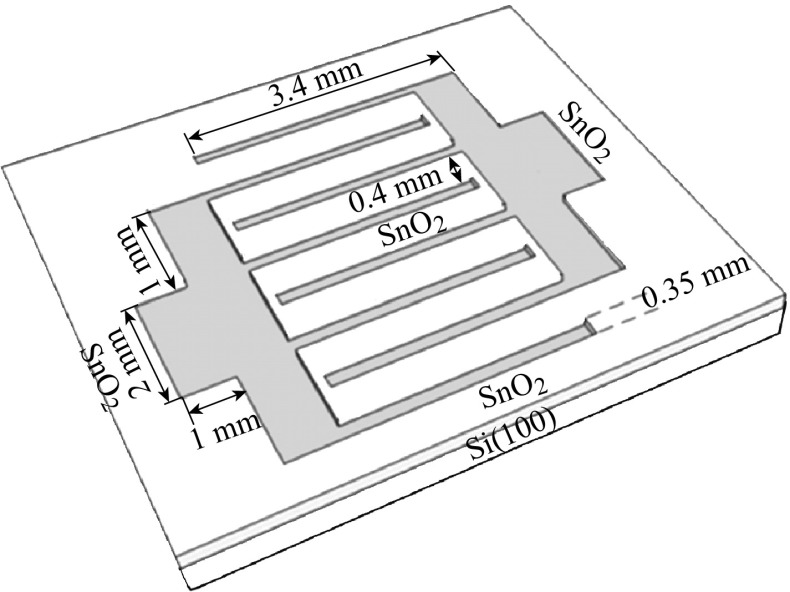
Schematic of Pd grid contact deposited on the nanocrystalline SnO_2_ thin films

The fabrication of the metal–semiconductor–metal (MSM) gas sensing devices was conducted via RF sputtering of Pd grid using a shadow mask on top of nanocrystalline SnO_2_ thin films. This mask contains two electrodes and each electrode consists of four fingers, the space between two neighboring fingers is 0.4 mm, and the width of each finger is 0.35 mm as shown in Fig. 1.

Gas sensing was performed in a homebuilt gas chamber, which was fabricated by an acrylic plastic box and heated by a small ceramic heater that joined to a temperature controller. The chamber was joined to three mass flow meters/controllers: the first for 0.1 % H_2_/balance N_2_ gas, the second for the carrier N_2_ gas, and the third for air ambient that is provided by an air pump via a drying tube containing fine silica gel. These mass flow meters/controllers used to supply a constant flow (1000 sccm) during test measurements. In this case, the resulted concentration of H_2_ gas (*c*_2_: in ppm) was computed by the following eq. [18, 21] *c*_2_ (ppm) = (*c*_1_ × *F*_H_)/*F*_total_, where *c*_1_ is the concentration of H_2_ in the bottle (0.1 % = 1000 ppm), *F*_H_ is the gas flow of 0.1 % H_2_ balanced with N_2_, and *F*_total_ is the total flow of (0.1 % H_2_/balance N_2_) and the flow of diluting N_2_ gas. The relative sensitivity (*S*) of the nanocrystalline SnO_2_ thin films gas sensor can be defined as the relative change in the conductivity ($\Delta G$) upon exposure to H_2_ gas [22, 23]:

1 $$S\left( \% \right) = \frac{\Delta G}{{G_{\text{air}} }} \times 100 = \frac{{\left( {G_{\text{g}} - G_{\text{air}} } \right)}}{{G_{\text{air}} }} \times 100$$

where *G*_air_ is the conductivity in an air ambient and *G*_g_ is the conductivity in the presence of the gas being sensed.

Also the relative sensitivity (*S*) could be rewritten in terms of the electric current generated in the semiconductor-based gas sensor on the application of a constant bias voltage [24, 25]:

2 $$S\left( \% \right) = \frac{{\left( {I_{\text{g}} - I_{\text{air}} } \right)}}{{I_{\text{air}} }} \times 100$$

where *I*_g_ is the current measured in the presence of the gas being sensed and *I*_air_ is the current measured in air ambient.

The crystal structure of the fabricated samples of the SnO_2_ thin films was performed using X-ray diffraction (XRD) analysis of PANalytical X’ pert Pro MRD equipped with a Cu Kα radiation of (*λ* = 0.154060 nm). The morphologies were characterized by field emission scanning electron microscopy (FESEM) of model Leo-Supra 50VP, Carl Zeiss, Germany. A current source (2400 Source Meter, Keithley, Cleveland, Ohio, USA) that was joined to a computer through the LabTracer (test integration software) was utilized to measure the electrical current passing in the gas sensing device on the application of a bias voltage.

## Results and Discussion

**Fig. 2:**
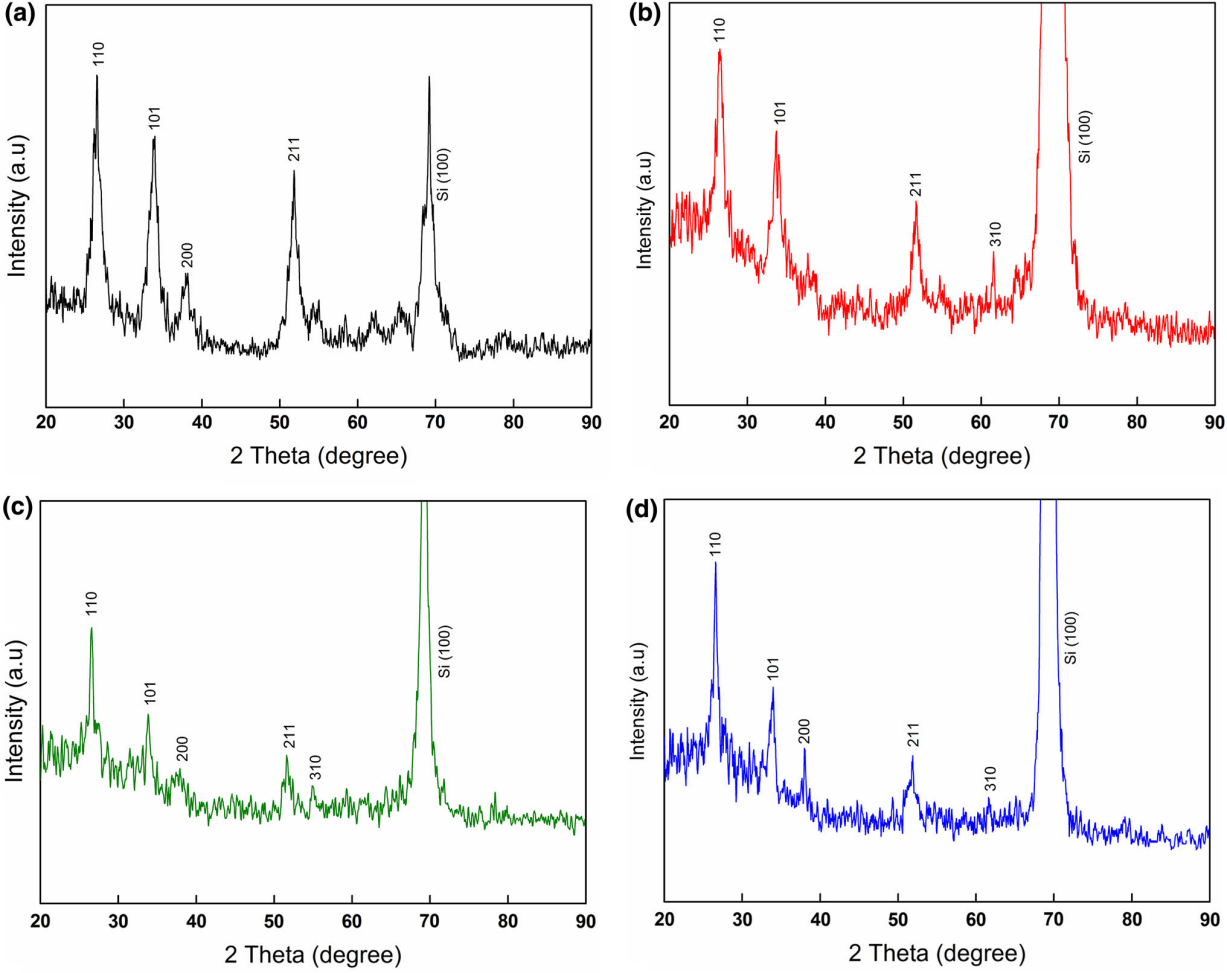
XRD patterns of nanocrystalline SnO_2_ thin films annealed at 500 and 400 °C **a**, **b** without glycerin, and **c**, **d** with glycerin

Figure 2 shows XRD patterns of nanocrystalline SnO_2_ thin films grown on bare Si (100) substrates, in which Fig. 2a, b are for films without adding glycerin and annealed at 500 and 400 °C, respectively. The peaks correspond well to standard bulk SnO_2_ with a tetragonal rutile structure (JCPDS card No. 041-1445) [26, 27]. While the peaks in Fig. 2c, d become sharper and stronger after adding glycerin to sol solutions. This is due to the annealing process which enhanced the crystallization of films, and therefore increased crystallite size, and reduced defects [27, 28].

The average crystallite size (*D*) of nanocrystalline SnO_2_ thin films was calculated using the (110) major and first diffraction peak by Debye-Sherrer formula [7]:

3 $$D = \frac{0.9\lambda }{\beta \cos \theta }$$

where *λ* = 0.1540 nm, *β* is the full width at half maximum intensity of the distinctive peak, and *θ* is the Bragg’s angle. It can be seen that the crystallite sizes increase after addition of glycerin as shown in Table 1.

**Table 1:** Crystallite size of SnO_2_ thin films obtained under different conditions

Case of glycerin volume ratios	Aging heat times at (70 °C)	Annealing temperature (°C)	Crystallite size (nm)
Glycerin volume ratio (0:1) (Glycerin-free)	8 h	500	28.35
	10 h	400	28.10
Glycerin volume ratio (1:12)	8 h	500	33.19
	10 h	400	33.20

**Fig. 3:**
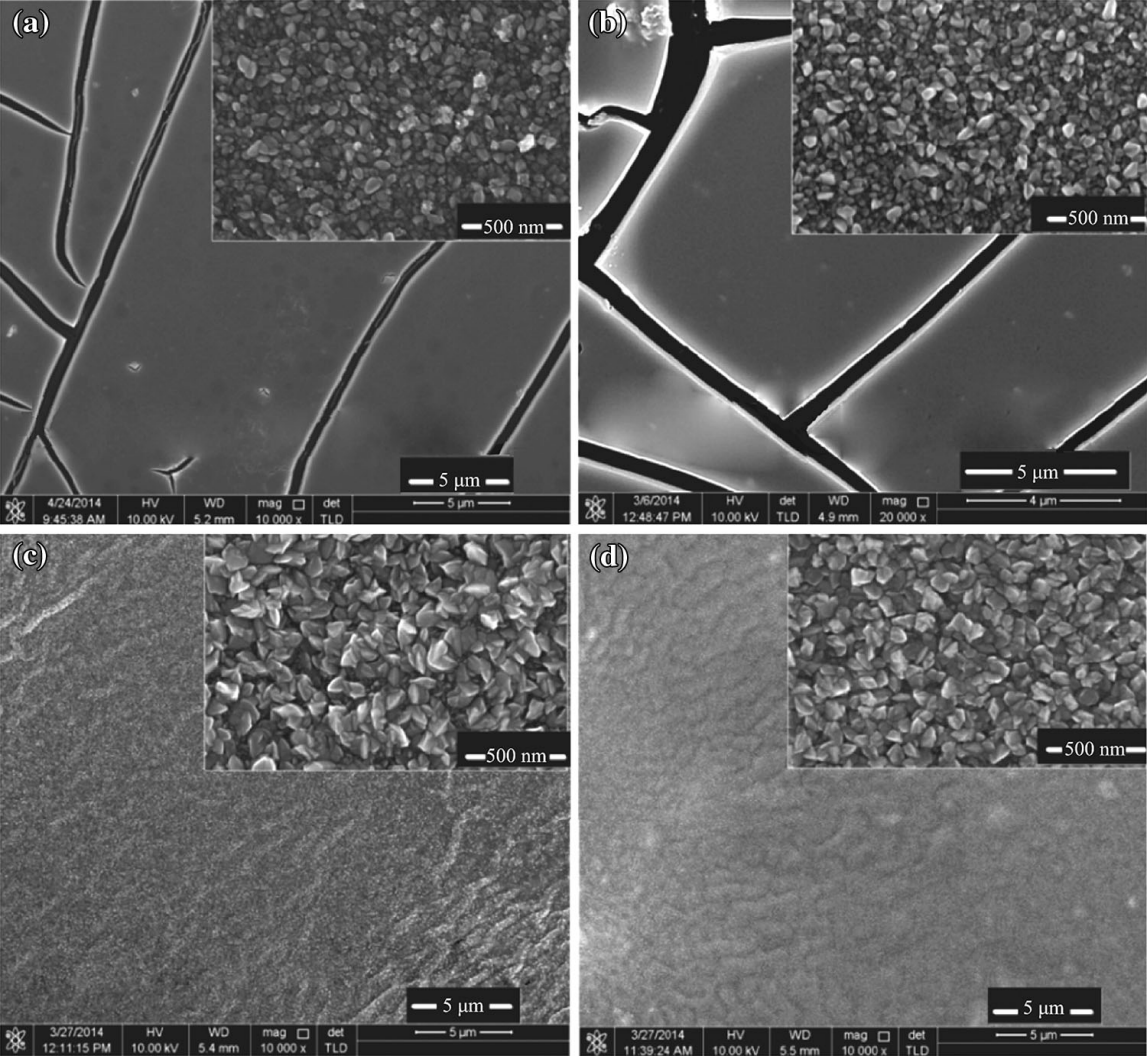
FESEM images of nanocrystalline SnO_2_ thin films annealed at 500 and 400 °C **a**, **b** without glycerin, and **c**, **d** with glycerin

FESEM images clearly show the morphology evolution of the SnO_2_ film grown on Si substrates with and without adding glycerin. Figure 3a, b show images of films without adding glycerin and annealed at 500 and 400 °C, respectively. One can notice the existence of cracks on the film. These cracks can produce negative effect on the performance for any device [7, 29]. Small nanoparticles with irregular size were observed to confirm the polycrystalline structure of the films. However, as shown Fig. 3c, d, there are no any cracks observed for nanocrystalline SnO_2_ films. This is due to the contribution of glycerin added to the sol solution with a volume ratio of 1:12 [7]. In addition, the particle size increases and becomes more regular and homogeneous. Figure 3c shows the surface morphology under the first aging heat time of 8 h at 500 °C. It is better than the second aging heat time of 10 h at 400 °C (Fig. 3d). This can be understood that increase of annealing temperature could enhance the crystallization degree of the films [27, 28].

**Fig. 4:**
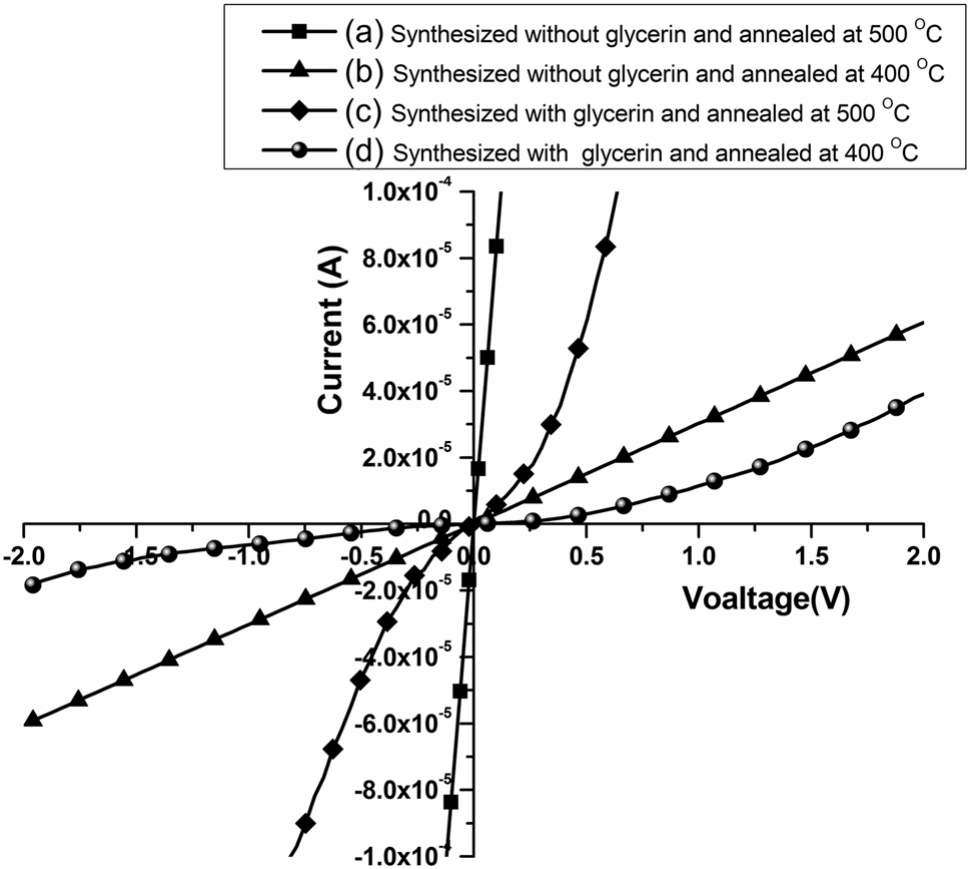
The *I*-*V* characteristics of nanocrystalline SnO_2_ thin films annealed, respectively, at 500 and 400 °C (a, b) without glycerin, (c, d) with glycerin, respectively

Figure 4 shows the *I*-*V* characteristics of nanocrystalline SnO_2_ films sensors, in which Fig. 4a, b show Ohmic behaviors, due to allow for contact between electrodes and bare Si substrates, whereas Fig. 4c, d show Schottky contact as a result of the work function for Pd element is higher than for SnO_2_ [30, 31].

**Fig. 5:**
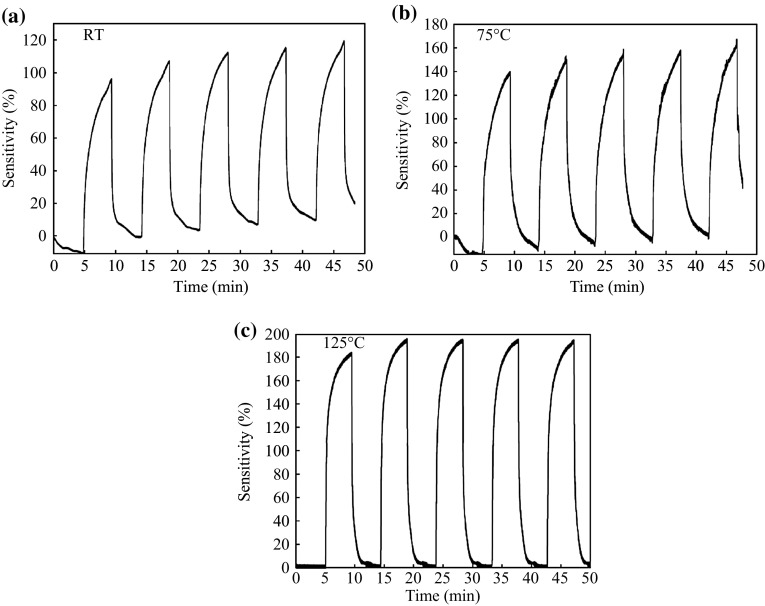
The sensitivity and repeatability of gas sensor based on nanocrystalline SnO_2_ thin films upon exposure to successive pulses of 1000 ppm H_2_/N_2_ gas and dry air at different sensing temperatures **a** RT, **b** 75 °C, and **c** 125 °C. The films synthesized with adding glycerin, aging 8 h, and annealing at 500 °C

**Fig. 6:**
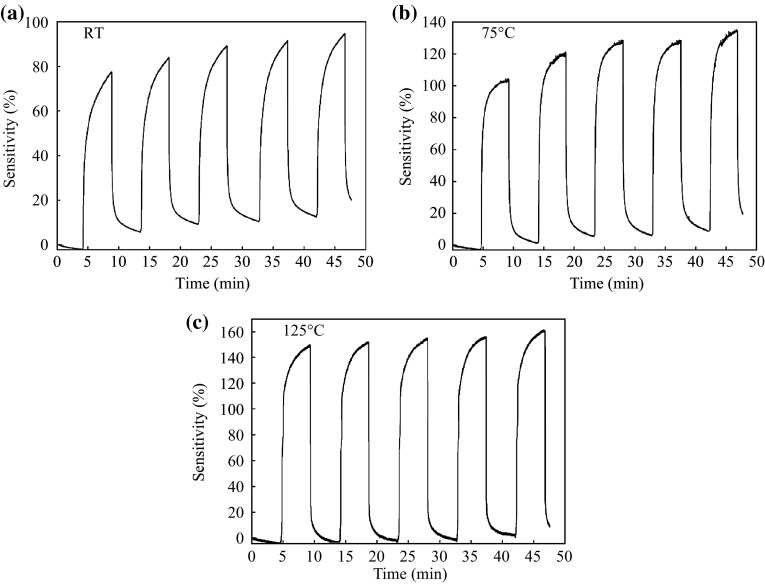
The sensitivity and repeatability of gas sensor based on nanocrystalline SnO_2_ thin films upon exposure to successive pulses of 1000 ppm H_2_/N_2_ gas and dry air at different sensing temperatures **a** RT, **b** 75 °C, and **c** 125 °C. The films synthesized with adding glycerin, aging 10 h, and annealing at 400 °C

The sensitivity (*S*) of the sensors with a Pd–nanocrystalline SnO_2_–Pd (MSM) configuration is denoted by Eq. (1). For Schottky behaviors, the sensitivity emerged at different temperatures (RT, 75, and 125 °C) and at different H_2_ gas concentrations, while there is no sensitivity on the sensors with Ohmic behaviors. The sensitivity of the sensors measured under different conditions are shown Fig. 5 (Device 1: 8 h at 500 °C) and Fig. 6 (Device 2: 10 h at 400 °C), where the exposure pulses is 1000 ppm H_2_/balance N_2_ and dry air, as well as the bias voltage is 0.2 V and 1 V, respectively. From Fig. 5a, the RT sensitivity of nanocrystalline SnO_2_ thin film sensor was found to be 120 %. It drifts from zero level during the repeated cycling of H_2_/balance N_2_ and dry air. This may be due to incomplete removal of H_2_ gas on the surface of nanocrystalline SnO_2_ films and the inefficient adsorption of O_2_ during the dry air injection into the gas chamber [32]. In addition, the sensitivity and repeatability increase with increasing the operating temperature are shown in Fig. 5b, c, respectively. The increases may be attributed to the increase of the adsorption/desorption of gas molecules in presence of different oxygen species [33].

**Fig. 7:**
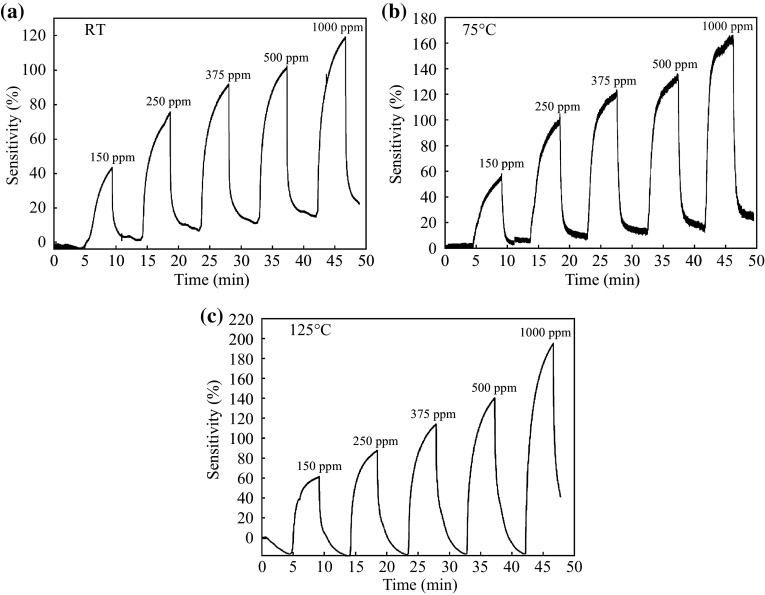
The sensitivity of gas sensor based on nanocrystalline SnO_2_ thin films under various H_2_ gas concentrations (150–1000 ppm) at different sensing temperatures **a** RT, **b** 75 °C, and **c** 125 °C. The films synthesized with adding glycerin, aging 8 h and annealing at 500 °C

**Fig. 8:**
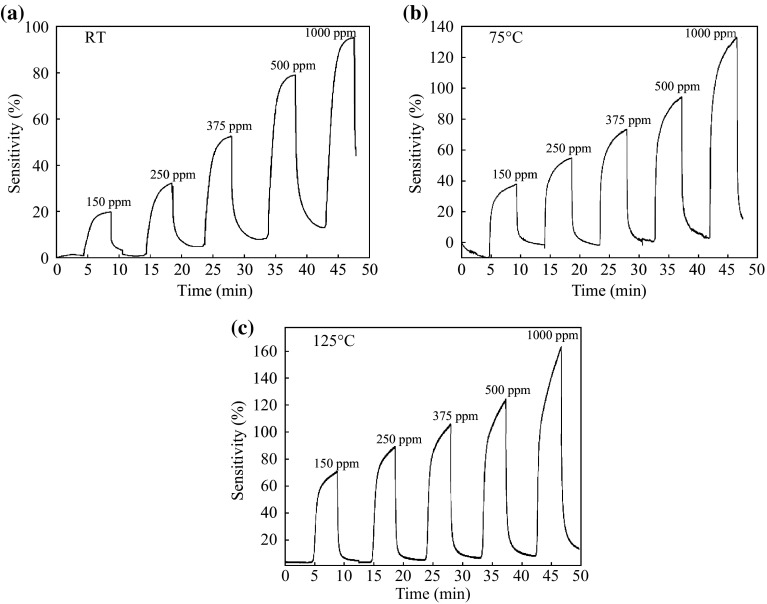
The sensitivity of gas sensor based on nanocrystalline SnO_2_ thin films under various H_2_ gas concentrations (150–1000 ppm) at different sensing temperatures **a** RT, **b** 75 °C, and **c** 125 °C. The films synthesized with adding glycerin, aging 10 h, and annealing at 400 °C

It is clearly observed that the sensitivity of Device 1 outperforms better than the latter as shown in Fig. 6, where the sensitivity value was 95 % at RT. This variation in the sensitivity value is due to the improvement of crystallization, which allowed for increasing in the electron–hole transport, and therefore the response to H_2_ increases [34]. The computed power consumption of two SnO_2_ films sensors is respective 65 and 86 µW for the detection H_2_ at RT. This makes that these sensors are easy to use in remote area where the available power may be limited. A significant variation in the sensitivity value of nanocrystalline SnO_2_ thin film sensors was observed upon exposure to H_2_ gas concentrations from 150 to 1000 ppm at different operating temperatures (shown in Figs. 7 and 8). Furthermore, one can see in Fig. 7a, b the sensitivity drift from the baseline. This is due to both incomplete recovery of H_2_ gas sensors and inefficient adsorption/desorption of the gases when the device is operated at low temperature compared with the operation of the H_2_ gas sensor at high temperatures (see Fig. 7c) [33]. Figure 8 displays the sensitivity value for Device 2 which is less than that of Device 1 at different H_2_ gas concentrations, which agree to the results in Fig. 6. The response time of H_2_ sensing is denoted as the time required to the final saturation state current was 90 % from its top value, while the recovery time is denoted as the time required to 10 % value of the saturation state current [33]. Table 2 displays the response and recovery times of nanocrystalline SnO_2_ thin film (Device 1) for the H_2_ gas sensing and their relative sensitivities at different operating temperatures were then compared with the previous studies.

**Table 2:** Performance comparison of nanocrystalline SnO_2_ thin film sensor (Device 1) with previous studies

Temperature (°C)	Response/recovery time (s)	Sensitivity (%)/H_2_ concentration (ppm)	Notice/references
RT	214/51.5	120/1000	This work
RT	220/–	60/20,000	Nanobelts SnO_2_ [39]
75	182/108.9	165/1000	This work
125	102.5/39.7	195/1000	This work
200	5–7/25–30	39/1500	Nanocrystalline SnO_2_ [40]
250	–/–	96/2500	SnO_2_ powder [41]

Figures 5, 6, 7 and 8 show a clear increase of sensitivity with high stability of nanocrystalline SnO_2_ thin film sensors as the operating temperature increases. This behavior is because of an enhancement in the adsorption/desorption processes of gas molecules, where there exist different types of oxygen species at higher operating temperatures [33]. Thereby, the reaction probability between oxygen species and H_2_ gas will be increased. Thus, the removal of H_2_ gas from the surface of nanocrystalline SnO_2_ thin film sensors is enhanced. As a result, it could be observed that the sensitivity is not drifted from the baseline.

The sensing mechanism of nanocrystalline SnO_2_ thin films is related to the reduction of the exposure to gas like H_2_ by the adsorption of oxygen molecules on nanocrystalline SnO_2_ surface. When the surface of nanocrystalline SnO_2_ thin films is exposed to air ambient, the oxygen species will be adsorbed. Depending on the operating temperatures, the adsorbed oxygen species will capture electrons from nanocrystalline SnO_2_ thin films surface and become negatively charged, which will increase the depletion region and therefore increase the resistivity [35]. There are different oxygen species depending on the operating temperatures, which can be described as follows: [36].

4 $${\text{O}}_{ 2} \left( {\text{gas}} \right) \to {\text{O}}_{ 2} ({\text{ads}})$$

5 $${\text{O}}_{ 2} \left( {\text{ads}} \right){\text{ + e}}^{ - } \to {\text{O}}_{ 2}^{ - } \left( {T < 100^\circ {\text{C}}} \right)$$

6 $${\text{O}}_{ 2}^{ - } \left( {\text{ads}} \right){\text{ + e}}^{ - } \to 2{\text{O}}^{ - } (T = 100 - 300^\circ {\text{C}}).$$

H_2_ molecules are dissociated to H atom on the Pd contact, which diffused to the surface of nanocrystalline SnO_2_ thin films, and reacts very quickly with different adsorbed oxygen species by negative charges [33]. Thereby the electrons captured by the oxygen species will return back to the conduction band of thin film, resulting in an increase of electron concentration in the conduction band so that the resistance of nanocrystalline SnO_2_ will reduce. The sensing reactions could be explained by using the following chemical reactions: [37, 38].

7 $$2{\text{H}}_{2} + {\text{O}}_{2}^{ - } ({\text{ads}}) \to 2{\text{H}}_{ 2} {\text{O}} + {\text{e}}^{ - }$$

8 $$2{\text{H}}_{2} + {\text{O}}^{ - } ({\text{ads}}) \to 2{\text{H}}_{ 2} {\text{O}} + {\text{e}}^{ - } \left( {T = 100 - 300^\circ {\text{C}}} \right)$$

9 $$4{\text{H}} + {\text{O}}_{2}^{ - } ({\text{ads}}) \to 2{\text{H}}_{ 2} {\text{O}} + {\text{e}}^{ - } .$$

The sensitivity of H_2_ gas sensor will reduce when the nanocrystalline SnO_2_ thin films are exposed to air ambient again, where the air ambient inputs to the gas chamber containing oxygen species. Thereafter, the air oxygen will react with the chemisorbed H_2_ on the surface of nanocrystalline SnO_2_ thin films. Hence, the resistance of the nanocrystalline SnO_2_ thin films goes back to its initial value [33].

## Conclusions

 Nanocrystalline SnO_2_ thin films were grown on bare Si (100) substrates using a simple cost-effective sol–gel method. The cracks that appeared on the surface of thin films were avoided by adding glycerin to the sol solutions in volume ratio of 1:12. Two devices with Schottky contact were fabricated to detect H_2_ gas with different concentrations and different temperatures. One of the devices shows a stable sensitivity of 120 % at RT with power consumption of 65 µW, which is appropriate in remote regions. The good sensitivity is attributed to the high porosity of nanocrystalline SnO_2_ thin film generated by adding glycerin. It makes easy for the adsorption/desorption of gas molecule. Moreover, Pd finger contacts significantly enhance the sensing properties of the gas sensor. The results also show that the good crystallinity of thin film can enhance the performance of device.
